# Understanding perceptions on 'Buruli' in northwestern Uganda: A biosocial investigation

**DOI:** 10.1371/journal.pntd.0006689

**Published:** 2018-07-30

**Authors:** Georgina Pearson

**Affiliations:** 1 Department of International Development, London School of Economics and Political Science, London, United Kingdom; 2 Population Health Research Institute, St George’s, University of London, London, United Kingdom; Kwame Nkrumah University of Science and Technology, GHANA

## Abstract

**Background:**

An understudied disease, little research thus far has explored responses to Buruli ulcer and quests for therapy from biosocial perspective, despite reports that people seek biomedical treatment too late.

**Methods and findings:**

Taking an inductive approach and drawing on long-term ethnographic fieldwork in 2013–14, this article presents perspectives on this affliction of people living and working along the River Nile in northwest Uganda. Little is known biomedically about its presence, yet ‘Buruli’, as it is known locally, was and is a significant affliction in this region. Establishing a biosocial history of ‘Buruli’, largely obscured from biomedical perspectives, offers explanations for contemporary understandings, perceptions and practices.

**Conclusions/Significance:**

We must move beyond over-simplifying and problematising ‘late presentation for treatment’ in public health, rather, develop biosocial approaches to understanding quests for therapy that take into account historical and contemporary contexts of health, healing and illness. Seeking to understand the context in which healthcare decisions are made, a biosocial approach enables greater depth and breadth of insight into the complexities of global and local public health priorities such as Buruli ulcer.

## Author summary

Buruli ulcer, a neglected tropical disease, has been described as an emerging public health problem in parts of sub-Saharan Africa. One of the challenges highlighted by the World Health Organisation (WHO) is improving access to biomedical healthcare. A research priority is thus to determine local understandings of skin disorders such as Buruli ulcer, and social-cultural factors that influence health-seeking. This article explores perspectives on Buruli among fisherfolk in northwestern Uganda along the River Nile, where the ulcer has previously been documented. The findings are based on a long-term ethnographic study of health, healing and illness in this region, and integrate insights from biomedical and social sciences. This biosocial approach demonstrates that, rather than seeking therapy late, people in this region sought treatment from local herbalists promptly when signs of skin lesions appeared. This was not because of non-biomedical understandings of disease. The reasons why people continue to trust local herbalists as experts in Buruli can be found in the historical context of how ulcers have been understood and managed, and the broader context of quests for therapy in this region. While the findings relate to where this study took place, the lessons learnt and biosocial approach used could be usefully applied in other settings where Buruli ulcer is endemic, and for understanding the local context of other neglected diseases and global health priorities.

## Introduction

A so-called neglected tropical diseases, Buruli ulcer occurs in rural areas with limited access to safe water, basic medical care and education [1]. Caused by *Mycobacterium ulcerans*, similar to mycobacterium that cause leprosy and tuberculosis, people affected develop nodules and other skin lesions typically on exposed areas of the body [1,2]. It is estimated that one third of early nodules resolve spontaneously [3]. However, severe long-term complications can develop from the toxin-producing bacteria with aggressive lesions causing permanent damage such as scarring, contractures and destruction of underlying bones. Outbreaks have occurred following ecological changes [4], and while the mode of transmission is not entirely clear, it has been associated with practices such as farming in swampy areas and along slow-moving water bodies, and various hygiene and wound care techniques [1,2,5,6]. The complexities that are not adequately understood through biological and epidemiological research clearly underscore the entanglement of biological and social dynamics of transmission.

Following a visit to West Africa in 1997, Dr Hiroyoshi Nakajima, then Director General of the World Health Organisation (WHO), declared Buruli ulcer an emerging disease and the Global Buruli Ulcer Initiative was launched in 1998 under WHO directive. Rather than interrupting transmission, which is poorly understood, the public health control strategy focuses on early detection and access to biomedical treatment [7]. However, there are implementation challenges. For instance, while the gold-standard is laboratory diagnosis, in practice a clinical diagnosis is often made based on presentation and exclusion of other skin diseases. And while the current recommendation for managing early lesions is an eight week course of antibiotics used in tuberculosis treatment and for severe lesions includes surgical intervention (amputation of the limb and skin grafting), the disease tends to occur in remote, rural areas where these facilities remain lacking.

Scope for control is further limited by paucity of data representing burden and patterns of disease. Reporting to the WHO has been in place since 2002, but evidence from West Africa where Buruli is highly endemic suggests significant under-reporting. In 1999, a national case search in Ghana identified 5619 patients with Buruli lesions (3725 active lesions and 1894 healed lesions) [8]. This is a substantial number given that from 2002 to 2012, between 2632 and 5867 cases were reported annually for Africa as a whole. Likewise in Nigeria, which typically has not reported cases, significant numbers were found in a case-finding study [9]. Epidemiological and clinical studies have thus pursued case-finding and mapping exercises.

Furthermore, it is often reported that people present late for biomedical treatment, when severe ulcers have developed. Thus, one aim set by the WHO was to: “reduce the proportion of category III [disseminated/severe] lesions from the 2012 average of 33% cases to below 25% by the end 2014 (*sic*)” [7, p.2]. Evaluations of access and barriers to biomedical healthcare report problematic healthcare seeking behaviours [10] providing explanations including stigma [11], knowledge and non-biomedical explanations of disease and forms of healing [12]. Yet, there has been little social science and anthropological research on this affliction. Noticeable exceptions include Grietens and colleagues’ [13] research unpicking the oversimplification between the role of local beliefs and treatment-seeking for Buruli in Cameroon, foregrounding an exploration of local knowledge. Whereas Giles-Vernick and colleagues [14] demonstrate additional insights to biomedical models of Buruli transmission by exploring ethno-ecological histories in Cameroon. More recently, anthropological insights have been incorporated into developing models for improving Buruli ulcer policy in Cameroon [15].

Northwestern Uganda is an intriguing area to study the historical and contemporary significance of Buruli ulcer. Historically, Buruli was significant in the region of northwestern Uganda along the River Nile. Ulcers were described in Uganda and Zaire (now Democratic Republic of Congo) by Sir Albert Cook in 1897 and Kleinschmidt in the early twentieth century [3]. Interestingly, a study of genetic diversity in Africa reports two main strains of Buruli with different lineages [16]. The Ugandan strain was one of the oldest in Africa (present for centuries), while the second strain (common in Gabon, Benin and Cameroon) appears to have been introduced in the 19th century–which the authors attribute to the upheaval of the neo-imperialism period. While Buruli primarily occurs in African countries, it was in fact first identified as Bairnsdale ulcer in Australia in 1948 and re-named from 1961 when many cases were reported in the then Buruli District (now Nakasongola District) of Uganda near Lake Kyoga [17,18]. During the 1960s and 70s, the Uganda Buruli Group described the disease among recent Rwandan refugees who settled near the River Nile [19]. At a similar time, Barker [17, 20] also associated the disease with swampy areas along the Nile, suggesting that the disease spread and became endemic in areas of Uganda after floods in 1962–64 created new sites of permanent swamps. Contemporary reported data suggests Buruli has declined as a public health concern in Uganda. In 2002, Uganda reported 117 cases of Buruli ulcer to the WHO (2632 from Africa, 3269 globally) compared to three in 2009 (5029 from Africa, 5084 globally) [21]. Since 2010, Uganda has not reported data, but previously the numbers were relatively low.

However, as literature from West Africa suggests, we clearly cannot rely on reported healthcare data to represent presence of disease let alone estimate prevalence [8,9].

This became evident during long-term inductive, iterative, ethnographic fieldwork in Moyo and Adjumani districts, northwestern Uganda, in 2009 and 2013–14, which this article is based on. In the two districts, while largely unseen at the health centres, Buruli ulcer was well-known among people along the river. What explains these diverging accounts?

This article addresses this question, taking as its starting point the perspectives of people vulnerable to (and affected by) neglected diseases such as Buruli ulcer. Echoing broader calls for biosocial approaches integrating insights from across the biological and social sciences [22, 23, 24, 25], this article builds on strands of interdisciplinary scholarship, bringing historical biosocial insight to understandings of afflictions and treatment practices. Drawing on long-term inductive, interdisciplinary fieldwork on neglected tropical diseases in Moyo and Adjumani districts in northwestern Uganda, this article presents an analysis of the social responses to an affliction known locally as ‘*Buruli’* (italics are used throughout the article to distinguish the vernacular use of the term, as opposed to the common biomedical name, Buruli ulcer which refers to the biologically defined *Mycobacterium ulcerans*), along this stretch of the River Nile. This suggests a more complex picture, and highlights the disparities between the framing of global health priorities and perceptions on these priorities by people affected. It challenges the oversimplification and problematisation of healthcare-seeking, and drawing on historical and contemporary insights, demonstrates the pragmatic and empiric nature of quests for therapy.

## Methods

This article is based on themes that emerged through long-term ethnographic fieldwork, in Moyo and Adjumani districts, Uganda, near the South Sudan border including three months of fieldwork in 2009, and a further 16 months in 2013–14. An inductive, iterative, interdisciplinary approach was adopted. The primary aim of the broader ethnographic research was to explore the context and everyday realities of neglected diseases among people living and working along the River Nile–a population deemed vulnerable to neglected tropical diseases–in order to understand the social responses to disease and public health, and through this establish wider implications for public health policy.

Ethnographic fieldwork included participant-observation, group discussions, semi-structured and open-ended unstructured interviews with key informants at fish landing sites (including fishermen, fish-processors, fishmongers, local council members, Beach Management Unit members and elders), along with health workers, local healers and district authorities (health and fisheries). The broader study included an epidemiological-parasitology survey conducted in collaboration with the District Vector Control Division of the Ministry of Health. This was of adults at twelve fish landing sites, one from each sub-county along the river and one island, investigating another neglected disease, schistosomiasis, (documented elsewhere [26]).This article presents findings that emerged from the ethnographic fieldwork related to Buruli ulcer, as documented in ethnographic fieldnotes, interviews and discussions.

Within this, twenty-one in-depth interviews were conducted with people who self-reported, incidentally in the survey and during further discussions and interviews, to have suffered from ulcers understood as *Buruli*. In other interviews and discussions, people discussed forms of local poisoning, some of which were related to skin ulcers. In-depth interviews were held with four male herbalists, identified by people locally, who treated suspected Buruli ulcer (*Buruli*), and numerous conversations including four semi-formal interviews were held with district health staff and healthcare workers on the subject. In addition, it was discussed in twelve semi-/in-formal group discussions with men and women at the landing sites. During conversations exploring understandings of ulcers and skin lesions, I sometimes showed people photographs from a poster (WHO, Global Buruli Ulcer Initiative), found in a health clinic cupboard. The photographs illustrated different forms of Buruli lesions, from nodules, papules and plaques, to oedema and various stages of ulcers. This elicited dynamic discussion on the causes of the different lesions as well as the types of ulcers that people had suffered or seen.

Discussions and interviews were held in either a local language–while I had some command of *Madi* to understand and participate in basic conversations, in-depth interviews were translated by an experienced local researcher who had conducted previous research with other research programmes- or carried out in English. Interviews were not recorded, but detailed notes taken and written up in full afterwards. Adults who had participated in the initial survey (not presented here) and follow-up interviews provided written consent. Rather than a one-off event, consent was an ongoing process and all adults who participated in additional interviews and discussions provided verbal consent. The London School of Economics and the National AIDS/HIV Research Committee (NARC) in Uganda for the Uganda National Council for Science and Technology (UNCST) granted ethical approval for the study (ARC141). UNCST granted research approval, and approval was also sought through both districts’ health offices.

This article presents an inductive thematic analysis of the ethnographic fieldnotes and interview transcripts. This was carried out manually, drawing out emerging themes on understandings of ulcers, healing practices sought and the relationship between local forms of therapy (*erua Madi*) and biomedical healthcare. These themes were strongly underpinned by a sense of historical legacies. Findings were triangulated with published data on Buruli and other ulcers in this region, by comparing the oral accounts and histories with available historical documentation, biomedical literature and other ethnographic accounts.

### Moyo and Adjumani Districts, Uganda

Moyo and Adjumani districts are situated along the River Nile bordering South Sudan. In the most recent census, 2014, the population of Adjumani was 225,251 (116,953 females, 108,298 males) and in Moyo was 139,012 (70,072 females, 68,940 males) [27]. The people are predominantly Madi (and identify as Christian, particularly Catholic, but also Protestant and, more recently, Pentecostal). However, in Obongi County of Moyo they identify with a number of neighbouring ethnic groups, including Lugbara or Kakwa related groups. A mix of languages are therefore used, but *Madi* is most widely spoken. The majority of people in Moyo and Adjumani are subsistence farmers, or fishing-farmers—along the river the land is particularly fertile and the fish business is important economically for men and women. People along the river are referred to as *meri ti ba* (people of the river) in *Madi*. It is difficult to assess the exact population along the river, with ongoing flux of people into and out of the fish business.

The northwestern region of Uganda has a long history of social upheaval, political and economic marginalisation [28,29]. Slave and ivory traders were active in the nineteenth-century, and subsequently the region was under various colonial and protectorate rule. During the colonial period, in now Moyo and Adjumani districts (then Madi sub district, named after the predominant ethnic group), there were a number of colonial public health campaigns, for instance for yaws and sleeping sickness. From the late 1970s, the time of decolonisation and Independence, through to the 2000s, there have been ongoing conflicts in the region causing an almost constant flux of refugee movements across the border with now South Sudan. During fieldwork, in December 2013, conflict broke out in neighbouring South Sudan leading to an influx of refugees into Adjumani and subsequently Moyo too.

Over time, people have experienced intermittent and long-standing presence of various NGOs including medical and humanitarian organisations. Aside from this, biomedical healthcare has largely been provided through both government and private facilities. Each district has a government hospital in the main town, which for some areas along the river is an hour’s motorbike ride away. Health centres at parish level are typically run by nursing staff and stock essential medicines. At sub-county level they also have some laboratory facilities. In addition, there are numerous private facilities, particularly drugstores, in trading centres. Thus in most areas along the river, there is some access to biomedical healthcare, although what is provided varies and is often limited. Alongside the public health system, there have been numerous disease-specific health programmes, for vaccine-preventable diseases, malaria and a number of the neglected tropical diseases. In contrast to other neglected diseases, such as intestinal helminths, onchocerciasis, human African trypanosomiasis and lymphatic filariasis, there has not to date been a specific public health programme for Buruli. Rather, the ‘strategy’ relies on patients self-presenting to the healthcare system—referred from the village health team or local health centre to a district or regional hospital (Arua, Gulu or Kampala). As well as biomedical healthcare facilities, therapies for various afflictions are provided by local herbalists and witchdoctors, as will be discussed in further detail below.

## Results/Discussion

Findings from fieldwork illustrate how in reality, people rarely presented to the health centre for suspected Buruli in the first instance. This was brought to my attention during a meeting early on in the research when a doctor pointedly asked, *‘but what do you know about Buruli ulcer*?*’* It was rarely seen in the hospital however ‘*deep in the village*, *it was found to be there’*. During fieldwork, it became clear that for people living and working along the river, *Buruli* was a well-known affliction. Unlike global health rhetoric, *Buruli* was far from emerging; and unlike at the hospital, it was far from unknown or unseen. While there had been some adoption of biomedical practices and understandings of the disease, people predominantly continued to use treatment that they knew and trusted from local herbalists. For other diseases, such as schistosomiasis, herbs were used as a ‘last resort’ when or where medicines were not available, and people made claims on their ‘rights’ to healthcare when these needs were not being met. Yet for Buruli, interestingly this was not the case.

For instance, at the time of fieldwork, one health centre near a fishing site was treating an adult with active Buruli ulcer while another reported to have seen a case in the previous year, but otherwise it was rarely diagnosed. On the other hand, during fieldwork, local herbalists near the river reported seeing cases of *Buruli* every year or two. One even suggested he saw somewhere between two to seven cases per year, including in the previous twelve months. This leads on to the questions: how can we explain these diverging accounts? How, and to what extent do understandings of *Buruli* relate to Buruli ulcer? How, and why, do people seek particular therapies for suspected Buruli ulcer, or *Buruli*, and what does this mean for public health policies seeking to address neglected diseases such as Buruli ulcer?

In the following, the findings from the analysis are synthesised around three inter-related themes: the historical context of Buruli ulcer in Moyo and Adjumani districts; understandings of ulcers known as *Buruli*, and their relation to other forms of skin disease and afflictions; and *erua Madi* (Madi medicine) and biomedicine.

### Buruli in historical context

A review of various field studies carried out in the 1960s and 70s in now Moyo and Adjumani districts shows that Buruli ulcer was a significant public health concern [17, 19, 30]. Clancey and colleagues [19] reported four active cases from Moyo; and Lunn et al [30] reported ‘burnt out cases’ suggesting earlier infection that had resolved. While Barker wrote: “on the west side of the river [Moyo], where the land is hilly, the disease is confined to the river’s edge; but on the east side [Adjumani], where the land is flatter, the disease extends up to 10 miles from the river” (p.43) [17]. He proposed that there was no evidence to suggest the onset of Buruli from more than 10 years prior to the 1962 flood (p.872).

The findings presented in this article reflect this time period—a number of adults interviewed were born in the early and mid-twentieth century. Those who reported to have had *Buruli* were affected from the 1960’s onwards, with the highest proportion affected in the 1990s. Yet, oral accounts suggest that these forms of ulcers clearly had a much longer history. Most people we spoke to, including elders and herbalists who had grown up in Madi region during the 1950s and 60s, used the term *Buruli* (although ‘*lupi lupi*’, referring to a swelling, was also used and understood by other regional language groups). However, it seems that prior to this period, similar ulcers were known by other names. Lunn et al [30] noted the terms used for ulcers as ““*juwe okoro*” or “*bile okoro*”, meaning “the sore that heals in vain”” (p.278). A herbalist born in the 1920s recounted to me that when he was training, the wound that is now known as *Buruli* was previously called *macodo—*translated as ‘abscess’ in a Ma’di dictionary compiled in 1941 [31]. He described *macodo* as ‘*a boil which swells bigger and is more serious’*, reporting that it was treated in a similar way to *Buruli* today. Likewise, another herbalist, born in 1939, recalled:

*This was the same disease; the same treatment*. *There are two types of jue*: *jue [boil] and macodo [Buruli]*. *It was the medical people who brought the term ‘Buruli’; I can’t remember which year the name changed*. *When the name came*, *it was thought to be a different sickness*. *So people would go to the medical for ‘Buruli’ … [A woman from the village was] treated there at the medical and it went to losing their leg*, *and then the people started to say*, *‘but it is nothing but jue macodo*, *except the mzungus [Europeans] call it Buruli’*. *So those who saw the experience of this person here started going back to the herbalists*.

Other skin lesions (nodules, boils, ulcers and wounds) were differentiated from *Buruli* and literature and documents from the twentieth century confirm that various forms of ulcer were endemic in northern Uganda. In the early twentieth century, ulcers were a preoccupation for the British administration in Uganda. Vaughan [32], for instance, describes the moral panic stemming from the high rates of ulcers among Bugandans, likely misinterpreted as syphilis, a sexually transmitted infection. On the other hand, in northern Uganda, treatment campaigns were for the non-sexually transmitted form of the disease, yaws (p.138). In a 1927 sleeping sickness campaign in Moyo it was reported that there was a “great number of cases of Yaws and Ulcers that came up for treatment” and during two months they administered nearly 2000 injections for these “often repulsive” ulcers [33]. Interestingly, during fieldwork in 2013/14, an elder described a disease that ‘bent the bone’ (seen with yaws) which he differentiated from *Buruli*, reporting that it had not been seen for a long time. The term *Buruli*, adopted by Madi to encompass particular ulcers, was thus differentiated from other skin lesions. While the vast majority of the swellings and ulcers discussed and identified as *Buruli* had not been diagnosed bio-medically, as I will demonstrate, there are consistencies in how *Buruli* and Buruli ulcer are identified.

### Understanding ulcers

People’s experiences and understandings of *Buruli* resonate with biomedical understandings of Buruli ulcer, and the majority of people, although unsure, used ideas of worms (*obu*) to explain it. This may be related to the focus on ‘worms’ in contemporary public health campaigns, particularly controlling intestinal helminths through mass treatment campaigns to ‘at risk populations’ including fisherfolk. In 2009, when I first visited this area of Uganda, fishermen often lamented, *‘we are the rubbish pit for worms*!*’*

In 2014, a herbalist explained Buruli transmission:

*What I think is that there should be the worm which is got in muddy places when there is a lot of dew*, *where the germ* [during the interview, the herbalist corrected the English translation given and said ‘bacteria’] *survives*. *You go there*, *you are affected*. *Mostly it is along the big rivers*. *Up like this you don’t get so many people affected*.

Many supported the deduction that Buruli was found along the rivers or in muddy places. In this sense, it was very much associated with fishing areas and practices. One elder and Beach Management Unit member wondered if *Buruli* was acquired from fishermans’ tools used in the past, such as hooks and nails. A few people recounted how the disease came from Buruli district, as this 70 year old herbalist explained:

*What I have realised is that there is a clan in Western Uganda called Buruli. They used to suffer with this sickness. The story is that they washed this wound in the river and so possibly the worms travel in the water to here*.

*Possibly you get it like that; maybe from drinking the water*.

One hypothesis was that it was ingested by eating mudfish. When gutting mudfish, women reported seeing small white worms in the stomach or gills, which some deduced might be the cause of *Buruli*. This was supported by seeing small white eggs, presumed to be those of the worms, in the lesions when the herbalist treated *Buruli*. Interestingly, mudfish are found in the Nile and its smaller tributary rivers and in the rainy season men go with spears to these areas to catch the fish. Given that transmission of Buruli occurs along slow-moving water bodies, this may be an activity that makes men vulnerable to infection.

The empirical basis to these deductions is clear. Yet, as with scanty biological explanations there was still an air of uncertainty and many unknowns. One male elder herbalist reflected:

‘*Perhaps it comes from God*, *because otherwise I don’t know*’.

However, unlike in West Africa where magical-spiritual explanations appear to be much more prominent [11], in Moyo and Adjumani, these explanations did not appear to be as pervasive as one might expect. In contrast to witchcraft or local poisoning, herbalists were clear that *Buruli* could not be inflicted by another person.

Even in the case of severe, late ulcers, the majority of people identified these as *Buruli*, although the distinction was not clear cut. A minority reported that these ulcers can be caused by *erua hwe*, a form of local poisoning; however this was an exception rather than the norm. Herbalists reinforced that these lesions occurred when they had not been treated in time and denied they had ever been a form of witchcraft or local poisoning. However, one elder herbalist had this to say of late ulcers:

*This is that which has been reported late and the person treating is fearful and hasn’t done a good cut. That one is not really Buruli, if it is realised, if it is really late Buruli it will eat your flesh and bones. But here, where you see the flesh alone is taken away, that is caused by erua hwe*.

He went on to say that he had recently seen a woman with this form of affliction.

*Last month, a certain woman died of such erua hwe after stepping on it. When you wash the leg, the flesh falls off and only the bones are left. She died in hospital. … She also arrived late [to seek treatment]*.

On the other hand, others described more distinction between the different afflictions. For instance, a 70 year old herbalist reported:

*With poison*, *it will not cause a wound*. *It goes into the blood vessels and destroys the organs*.

Another 52 year old man who had *Buruli* when he was 14 reported that he had not seen late ulcer and when asked about local poisoning and *erua hwe*, replied:

*The one of erua hwe or others is quite different*. *It doesn’t bring wounds like this*, *but pain*.

In light of these different explanations, a 62 year old woman who had experienced *Buruli* in her 20s, reflected on how treatment informed the distinctions:

*Before [1960s] people would think it was erua hwe or inyinya and they were using drugs of those ones but they were not getting it [it was not healing]*. *And then they realised it was not these two and started to find other treatments*.

Other forms of ulcer-causing poisoning also existed in the past. One elder fisher-farmer showed a scar above his ankle from an ulcer when he was 18 years old, and explained:

“*I stayed down for two years with the wound. It was like that of Buruli, but was not Buruli. Local herbs were also applied to treat it. These herbs were antipoison, which you apply on the wound and also drink. Then you pass the poison that has reached inside out with diarrhoea. The herbalist uses different herbs to that of Buruli*.

*The one of Buruli, it collects and is cut. But this one, the whole leg itches and you can get paralysed. I know the difference between the two, because the son of my brother had Buruli. This one, the poison, is only cut superficially, not deep*.”

Thus, these accounts show how over time, ulcers and lesions have been differentiated by signs, symptoms and observed effectiveness of treatment, drawing on overlapping explanatory models.

Exploring perceptions, understandings and experiences of *Buruli* within this, when shown the photographs from the WHO poster all but one woman indicated that the nodule was *jue*, a boil. Seemingly, papules were rarely seen, with only one person suggesting it could be *Buruli*. On the other hand, people identified plaques, non-ulcerative oedema and early ulcers as *Buruli*. Rarely, early ulcer with indurated edges was associated with cancer. On the whole, most people who had had *Buruli* had experienced forms of early ulcer. On many occasions this wound developed after the herbalist began treatment. Some were affected once, others multiple times. Similar to epidemiological descriptions, the vast majority of people had experienced lesions on their legs which most commonly started with an itch, described as like a small prick of a thorn, developing within days into a small swelling or skin changes. Others first noticed a small nodule, and a minority experienced pain or other sensations.

Noticeably, people clearly recalled their experiences of *Buruli*. A 20 year old female fishmonger was affected when she was eight:

“*It started when I was going to fetch water*. *As I was walking*, *I felt at the buttocks itching*, *and started scratching*. *On the second day it started swelling*.*”*

Likewise, a 36 year old fisherman recalled:

*“I was in Primary school*, *in 1994*, *when I got this attack of Buruli ulcer on my right knee (he shows the scar of the ulcer and the herbalist’s cuts)*. *I was coming from school and got an itching as if something had bitten me on the knee*. *I was itching and scratching the spot and it became lighter*. *This carried on for four days*, *and it was experts* [herbalists] *at home who examined me*. *They touched it and said it was Buruli*.*”*

And a 28 year old female fishmonger recollected:

“*I suffered from Buruli in 2002*, *on my right leg*. *I first had that feeling as if I had sat on one leg for a long time*. *This was there for one or two days*. *Thereafter*, *the leg was paining and swelling [on the right thigh]*. *People around said it might be a boil*. *They took me to a herbalist who said it was* *Buruli*, *but he couldn’t treat Buruli so I was taken to another herbalist* …”

Not only do these narratives overlap with biomedical descriptions of Buruli, in addition, they demonstrate how quickly people sought ‘expert’ advice. Contrary to experiences at health centres, and perceptions on late presentation portrayed in public health literature, people in fact sought what they deemed appropriate healthcare relatively quickly. Advised by parents or other family members on where to seek treatment, people affected by ulcers, *jue* and *Buruli* largely sought therapy from a herbalist, and the majority did so within a few days of experiencing symptoms. Despite *Buruli* being understood to a large extent as a disease with biomedical causes, the *experts*, were local herbalists. It was the herbalists and the perceived efficacy of their treatment that people trusted.

Herbalists that treated *Buruli* learnt their skills, techniques and practices from elder herbalists. Their apprenticeship took years. Knowledge of herbs was learnt from their teacher, revealed in dreams, or through trial and error. While some herbs were collected from elsewhere, sometimes at the foot of a mountain, they were predominantly found growing wild around the home. One elder herbalist pointed to the common grasses around his house that he used, and laughing said, ‘*you see*, *these people don’t know’*. These local herbs are referred to as *erua Madi* (literally, ‘Madi medicine’), or *erua abi dri* more generally (‘medicine from ancestors’, or ‘medicine given by ancestors’) and are opposed to *erua Mundro* (‘European’s medicine’, or biomedicine).

When a herbalist was approached for advice he first assessed whether the lesion was *Buruli*, *jue* or another condition. One herbalist described examining for pitting oedema–observing an indentation after pressing the swelling with a thumb–a sign of *Buruli*. Another test involved making a cut at the site of the lesion (for example on the leg) and elsewhere (for example on the arm), demonstrating to the person that the colour of the blood from the area with *Buruli* was darker.

Herbalists’ treatment varied to a certain degree, but their general approach was similar. A 36 year old male fisher described his experience:

*It was four days from when the itching started to when the whole area became brown and paining inside. There was no swelling. As I kept scratching, the colour of the skin became light*.

*Within these four days, I immediately went to the expert. Straight away the herbalist cut me with a razor blade small, small [superficially] and applied local herbs. On day six, the herbalist said it was now ready to be cut deep and pus came out. Day six he cut three deep holes; day seven he checked it; day nine he cut four more deep cuts. He continued like this up and down [the leg]. The pus was coming from the wound on the knee, not the cuts to the leg. The cuts were made higher up so that the germ didn’t spread further*.

*It took one year and four months before I could walk*. *The wound almost took out my knee cap; it almost became stiff but because I was brave I kept moving the leg*.

Herbalists often began by making small superficial cuts to the skin around where the problem was ‘*to make the ‘germs’ collect in one place*’. Typically, after a number of days when the swelling had accumulated, the herbalist made a deep cut to release pus immediately around the swelling. Mixtures of fresh or dried herbs were then applied topically. In addition, some herbalists made a drink from the herbs. Others specifically did not use herbal drinks for *Buruli–*only for local poison as herbal drinks precipitate diarrhoea and expel poison from inside the body and bloodstream.

This treatment was sometimes extensive, especially considering anaesthetic was not used. As one woman described during a discussion with female fishmongers:

… *when I got it*, *it was so bad*. *I was nearly dying from the smell of the wound on my knee*. *My father said that they needed to treat it seriously*, *but that it would be so painful that I must be held down*. *But I explained that I didn’t need holding down*, *as I was nearly dying*.

Herbalists explained that in the past, such severe forms of *Buruli* requiring extensive treatment were more common. The deep cuts were completed in one sitting, as one herbalist said:

‘*You cannot be fearful to cut’*.

He explained that if it is not completed in one sitting, ‘*it will continue to eat the flesh’*, and the wound will spread, developing into a late ulcer. He went on to state that they had to be very careful managing the waste from cleaning the wound–‘*so that no one can tamper with it*’. Reflecting on how common it used to be for a number of people in one household to be affected, he suggested that perhaps this wasn’t done so rigorously in the past.

Depending on the severity, the wounds took weeks, months or years to heal. For a few people, *Buruli* had affected the use of the limb and it was common for people to suffer from pain long after the ulcers had healed, particularly when it rained. One male elder continued to make superficial cuts and apply herbs to his leg each period the rainy season began and his pain returned. *Buruli* had also affected some people’s day to day lives, limiting women’s ability to carry out daily chores, or children’s time spent in school. There were reports of people having died from late ulcer lesions, but most it seemed recovered and healed with little long-term sequelae except scarring, which people openly showed when the topic of *Buruli* came up during conversation. Some of these scars were the depressed scars of the ulcer itself, others were from the herbalist’s cuts.

### *Erua Madi* and biomedicine

Over time, there has been a continuing relationship with biomedicine surrounding treatment practices for *Buruli*. Along the river, some people who experienced *Buruli* had solely used *erua Madi*, with others using a combination of *erua Madi* and biomedical treatment. This reflects broader plurality in quests for therapy in this region. In part, decisions around treatment were influenced by who people went to for advice–normally a family member—before seeking treatment; and in part by their own experiences, or the circumstances that people found themselves in at the time. Only one 23 year old fisherman reported that he solely sought biomedical treatment–in this case, he was taken by his parents to a nearby health centre. Otherwise, people who had been affected, particularly those affected in the last 15 years, reported that they had initially visited a local herbalist for treatment (cutting) before attending a health centre for antibiotic injections. For instance, in 2013 a 30 year old woman first sought treatment from a local herbalist before concurrently receiving antibiotic injections and finally undergoing surgery. Another man suffered *Buruli* three times in the 1980s and 1990s. The first time he solely used *erua Madi*, however when the ulcer recurred he concluded that the local herbs had not sufficiently treated it and so sought biomedical treatment. At the second recurrence in the 1990s, during which time he was displaced due to conflict, he again used *erua Madi*.

It is noteworthy that both *erua Madi* and biomedicine can be used for *Buruli*. Contrastingly, for local poisoning only *erua Madi* can be used: if a person affected by local poisoning consumed *erua Mundro* their condition deteriorated or they experienced a potentially fatal reaction to the medicine. During an interview about *Buruli*, a 20 year old female fishmonger described a separate experience of local poisoning that was initially treated at the hospital:

*There was another serious sickness I had when I stepped on something [erua hwe] that pricked me. I was more than fifteen years old. I reported it to my parents as it was paining me. After three days it got worse and so they took me to hospital. I had my stool examined and was given drugs from the hospital. Our people say that if you take erua Mundro for local poisoning, it will increase. So it got worse … Within three days I experienced my head turning [she demonstrated a painful spasm]. When I stopped the hospital medicine and begun the local herbs, it resolved … So they took me to a local herbalist and I was treated for four months. They were cutting and applying local herbs, and I was taking a drink of herbs. This drink gave me lots of diarrhoea. There was no big wound to treat–only the pain*.

Describing this adverse reaction to the biomedicine administered in the hospital, this experience was understood to be a sign that therefore the cause of her initial sickness was not biomedical. Rather, it was a form of poisoning, and this explanation was reinforced by the fact that when the biomedicine was stopped and the herbalist’s treatment started, the reaction subsided. Thus, through these empirical observations herbalists were deemed best placed to deal with particular conditions. Yet seeking *erua Madi* from local herbalists was not solely because of non-biomedical understandings of disease and illness. While *erua Madi* was used for *Buruli*, in contrast to local poisoning this was not because it was an interpersonal affliction. *Buruli* was still a Madi disease, because of the history of ulcers and treatment pre-dating the introduction of biomedicine.

In the case of ‘worms’ like schistosomiasis, people often reported that they used *erua Madi* as a last resort, because they were far from a health centre, the health centre was regularly out of stock of medicines, or they were not able to access the free distribution of praziquantel. Research on social responses to mass drug administration for schistosomiasis and other neglected tropical diseases in Moyo and Adjumani has demonstrated how demand for the public health programme and biomedicine in part draws on ideas of modernity and questions of citizenship–a demand for the state provision of resources where it has typically been lacking [34]. Even though the public health provision of Buruli ulcer treatment is similarly limited, such claims questioning the lack of biomedical treatment are not being made. In part this could be due to limited public health campaigns in contrast to other diseases. For instance there have been numerous, long-standing malaria interventions, such as the distribution of bednets and provision of anti-malarial treatment and Co-artem for malaria is widely accessed and used as soon as symptoms interpreted as malaria develop. More recently, hepatitis ‘emerged’ as a serious public health concern in 2010. A childhood vaccination programme has been introduced, yet there is demand for biomedical testing, vaccination and treatment beyond this, and a questioning of the limited availability of these resources [34]. Social responses to *Buruli* have clearly evolved in a different way to these other diseases.

During long-term fieldwork in the 1980s, Allen [34] describes a very similar situation, with *Buruli* understood as a Madi illness from impersonal causes requiring treatment from herbalists. Standard antibiotics at the time would not have been the current WHO-recommended regime for Buruli ulcer, and Allen [34] similarly notes that despite complications and fatalities, some cases of severe disease were cured by herbalists. Allen points out that this provided further empirical support for their expertise. Even earlier in the twentieth century it appears to have been a similar picture. Lunn et al’s study of Buruli ulcer in Madi District in the 1960s found that out of 39 new cases of disease, “On two occasions patients were found to be applying powdered herbs, a procedure which forms a thick dry crust over the ulcer bed” (p.278)[30]. The authors also reported evidence of ‘burnt-out’ cases: “some parents displayed their children proudly, affirming that their ulcers had healed without Western medicine” (p.279)[30]. Thus, there has not been an apparent dramatic change in practices over this 50 year period, and the perceived efficacy of herbalists’ treatment has persisted despite the general expansion of biomedicine during this time [29].

To some degree this is not surprising and there are a number of possible explanations. On the one hand, there is a perceived lack of efficacy of biomedical treatment. There has not been a concerted public health strategy to actively identify and treat Buruli and, from what I could ascertain, the broad-spectrum antibiotics widely available at local health centres were not the WHO-recommended regime (drugs which were available through HIV-TB services). On the other hand, there is a perceived efficacy of herbalists’ treatment with successful treatment of many nodules and ulcers providing evidence. This raises the question: what is it about herbalists’ treatment and practices that are effective? Interestingly, it has been reported that one third of early stage Buruli nodules resolve spontaneously and excision is estimated to have an 84% cure rate [3]. As described in this article, people tended to seek herbalists’ advice within a few days of initial symptoms, and herbalists treated nodules at this early stage. Furthermore, in Ghana, antimicrobial properties have been identified in the herbs used and hot poultices applied [35, 36]. Therefore, there are conceivable biomedical explanations for the effectiveness of herbalists’ practices.

But the effectiveness of herbalists’ treatment and practices goes beyond a consideration of biomedical plausibility, nor has it been without biomedical influence. Herbalists have incorporated biomedical knowledge, and their practices have adapted to new developments, technologies and biomedical threats. One herbalist explained how their practices had changed. Before a period of exile in the 1980s when people fled to southern Sudan, herbalists made their cuts using the head of an iron spear. Since razor blades became available, patients are required to bring their own, and whereas before the blades were reused, now they are for sole use, as one herbalist explained:

‘*you can’t use the blade on this person*, *and then that one*: *you can’t pass on AIDS*’.

It was also not uncommon for herbalists to have a consultation record book, similar to hospital record-keeping, with details on the patient, sickness, and treatment given. There have been attempts to more formally professionalise herbalists [34] and herbalist associations have been established in the town. Yet the majority still practiced independently at their homes particularly in rural areas with little formal governance.

The relationship between herbalists and biomedical practitioners has not been without tensions. As a 70 year old male herbalist recalled:

*Three years ago I was treating someone here and a nurse came around and started quarrelling. She was quarrelling that I should not be treating these things and she said that she should take me to the authorities. But I said, ‘you take me, for if this person comes to you, you will wait and wait saying it is not ready yet, whilst inside it is rotting.’ Now later she came back and apologized, that they are meant to say these things. Now she advised me to do the cutting and then when it is finished with the cutting and just a wound, then I should send them to the health centre for antibiotics (he mimes sprinkling antibiotics on the wound), ‘PPF’ [an antibiotic, Procaine Penicillin Forte, commonly referred to], and dressing*.

While biomedicine was seen to offer antibiotics, as this herbalist explained, for *Buruli* it is the cutting and visible release of pus that is important, otherwise neither treatment will work. Cutting is a key feature of the herbalists’ treatment. This is interesting because some studies have suggested that a fear of surgery leads people to avoid hospital care and seek traditional healers [11, 37]. Yet from my discussion with people in Moyo and Adjumani, and seeing the scars of healed ulcers, a herbalist’s treatment was sometimes extensive, and treatment by a herbalist was preferred because of a perceived lack of intervention by medical staff at the health centre. People explained how the condition worsened if somebody attended the health centre as medical staff, following protocol, waited before lancing nodules or they only administered antibiotic injections. In one village, women described how healthcare workers ‘feared’ to lance a boil, preferring to refer patients to a herbalist (although the herbalist referred to denied being aware of this). In addition, antibiotics commonly available at health centres were likely not the most effective, depending on the microbial cause of the lesion. Furthermore, health centres’ drug stock is frequently limited, further undermining biomedicine as a credible source of treatment. On the other hand, *erua Madi* is empirically perceived as efficacious and the long history of herbalists managing *jue* and *Buruli* with *erua Madi* mean they are trusted as experts.

Thus, there are multiple reasons why people rarely presented through the biomedical health system–labelling this as ‘late presentation for treatment’ is clearly misleading. Village health teams were reportedly advised to report ‘wounds that don’t heal’ to health centre staff, and to ‘monitor and inform’ the health authorities of local herbalists treating such cases. But in reality, a healthcare worker explained:

“*Cases of Buruli only come to the district when they are necrotic; otherwise they use local herbs … People locally know there is no treatment in the hospital … so early diagnosis is a problem*.”

Indeed, during a conversation, one woman even asked me:

‘*but is there treatment at the health centre*?’

Herbalists raised similar concerns in the past about the need for early diagnosis and treatment, but now, it was said, people were aware that if they sought treatment from a herbalist early they could be cured within a few days without the need for deep cuts. From this perspective, there was little need to question a lack of biomedical input or even seek it, at least initially and certainly not solely. In this respect, rather than ignoring initial symptoms of *Buruli* and *jue*, people were responding quickly and seeking out treatment that they deemed appropriate (whether solely from the herbalist or a combination of *erua Madi* and biomedicine), even if that doesn’t fit biomedical notions of appropriate treatment.

Accordingly, there has been an apparent reduction in severe *Buruli*. As a 70 year old herbalist remarked:

*[Buruli] is practically getting finished; they might get rid of it one day*. *Because these days it rarely goes to deep cuts*, *only the small cuts*. *It has changed because you hardly can now get the big cuts*. *It is only the adults now*, *who were treated a long time ago*.

Another herbalist also reflected on the declining number of cases and clusters within households. Likewise, a district staff member reported that Buruli was ‘near eradication’, yet these patterns of disease were not documented, with limited, or no epidemiological data. It is not entirely clear what enabled this reported decline given that people are still involved in fishing and farming along the river where the disease is likely to be found.

*Buruli* remains significant though: these historical encounters shape contemporary responses. When I asked people along the river how they would advise others who developed *Buruli*, invariably the reply was ‘*to go to a herbalist*’. In this article I have described the situation in rural areas along the river, and responses may well be different in towns where people have access to the hospital and numerous private clinics. Indeed, when a young boy in a district town developed ‘*jue’* his mother took him without question to a nearby private clinic for lancing and antibiotics. However, when a young fisherman at one landing site developed a painful swelling on his leg he adamantly refused to attend the health centre, even when I offered a lift:

“*This is just jue; I will deal with it*, *I will cut it tonight at home*”.

Other people present confirmed: these sorts of afflictions, *jue*, *Buruli*, were treated at home not at the health centre.

Is Buruli ulcer a neglected disease in this region? Arguably, it depends on whose perspective is taken. Buruli ulcer remains a challenge: hospitals and health centres rely on people self-presenting, yet, for the reasons given people rarely do so. Far from being an ‘emerging public health concern’, for people living and working along the River Nile in Moyo and Adjumani, an area historically endemic for Buruli Ulcer, *Buruli* was a well-known affliction and similar conditions have been managed long before the introduction of biomedicine. It was neither emerging nor perceived as a particular threat.

### Conclusion

The accounts presented in this article clearly show how statements of late presentation for biomedical treatment and healthcare-seeking are misleading and oversimplified. This historical biosocial analysis of *Buruli* in northwestern Uganda has elicited alternative, and deeper insights into contemporary perceptions and practices. The significance of the history of ulcers, herbalists and biomedicine in this region was evident–an aspect often understudied. Piecing together published accounts and oral histories shows how biomedical and historical documents echo people's accounts; from both perspectives, ulcers have continuously been reinterpreted. Understandings of *Buruli* are not necessarily contradictory to biomedical models, and do not exclusively explain healthcare-seeking. Understandings of ulcers and treatment practices in this region have developed over a long encounter with skin lesions, shaped by historical and contemporary encounters with biomedicine and long-established therapies. This enquiry illustrates the pragmatic, empiric nature of quests for therapy, and the early presentation to herbalists who are trusted to manage these lesions based on the longevity of the afflictions and healing practices. Establishing this social history goes some way to explain contemporary responses to ulcers, healing and healthcare.

This research builds on calls for biosocial approaches to global health priorities including neglected tropical diseases, and anthropological insights into local beliefs and treatment-seeking. Thus far, research that has explored non-biomedical understandings of disease and therapies for Buruli ulcer has largely not considered the broader historical and social context. Taking this into account elicits additional insight into why people seek healthcare in particular ways. Exploring responses to *Buruli* in northwestern Uganda shows how these lesions are part of the history of this region and elicits insights into understandings of afflictions, quests for therapy and encounters with biomedicine, which bear relevance for understanding contemporary perceptions and practices relating to global and local public health priorities. However, there are limitations. Firstly, this article is based on an analysis largely of accounts of previous quests for therapy for *Buruli*/Buruli ulcer–it was not possible to follow quests for therapy as they unfolded, nor was it possible to say for certain if the self-reported cases were Buruli ulcer, although the oral accounts reflect biomedical accounts. Secondly, it was beyond the scope of the study to carry out diagnostics, and therefore we cannot document biological presence of disease. Finally, the impact of Buruli in Uganda is arguably not comparable to that presented in research from highly endemic countries such as in West Africa, however, there are broader insights gained from reflecting on the approach presented in this article.

That is, what a biosocial approach to understanding healthcare-seeking for Buruli ulcer might look like, and the insights that this type of approach can bring.

This analysis thus raises additional questions that cannot as yet be answered: What are the biological explanations for the lesions understood as *Buruli* in this region? If prevalence has reduced, how, and why, has this come about? To answer these questions will require further inquiry that encompasses biological, social, epidemiological, ecological, environmental and historical insights.
